# Treatment patterns of drug-naive patients with type 2 diabetes mellitus: a retrospective cohort study using a Japanese hospital database

**DOI:** 10.1186/s13098-019-0486-y

**Published:** 2019-11-01

**Authors:** Yohei Morita, Hiroki Murayama, Masato Odawara, Melissa Bauer

**Affiliations:** 1 Medical Division, Novartis Pharma K.K, Toranomon Hills, Mori Tower 23-1, Toranomon 1-Chome, Minato-ku, Tokyo, 105-6333 Japan; 2 Department of Diabetes, Endocrinology, Metabolism and Rheumatology, Tokyo Medical University, 6-1-1, Shinjuku, Shinjuku-ku, Tokyo, Japan; 3 Real World Data Analytics, Novartis Global Service Center, Vista Building, Elm Park Business Campus, Merrion Road, Dublin, Ireland

**Keywords:** Database analysis, Dipeptidyl peptidase-4 inhibitor, Drug-naive, Japan, Metformin, Real world, Treatment intensification, Type 2 diabetes mellitus

## Abstract

**Background:**

Guidelines for Type 2 diabetes mellitus (T2DM) management in Japan provide physicians the discretion to select treatment options based on patient pathophysiology of the disease. There exists a wide variation of preference for initial antidiabetes drugs (AD). The current database analysis aimed to understand the real world treatment patterns in drug-naive patients with T2DM in Japan.

**Methods:**

We analyzed data of patients (≥ 18 years) diagnosed with T2DM between October 2012 and September 2016 from the Medical Data Vision, a Diagnosis Procedure Combination database. The primary objective was to determine the proportion of T2DM patients receiving each type of treatment as first-line therapy among the drug-naive cohort.

**Results:**

Of the 436,546 drug-naive patients, 224,761 received their first-line T2DM treatment in the outpatient setting. The mean age of the patient population was 65.6 years at index date. Dipeptidyl peptidase-4 (DPP-4) inhibitor was the most prescribed (56.8%) outpatient AD monotherapy, followed by metformin (15.4%). DPP-4 inhibitors were prescribed over metformin in patients with renal disease (odds ratio [OR]: 4.20; p < 0.0001), coronary heart disease and stroke (OR: 2.22; p < 0.0001). Male (OR: 1.03; p = 0.0026), presence of diabetic complications [retinopathy (OR: 1.33; p < 0.0001), neuropathy (OR: 1.05; p = 0.0037), nephropathy (OR: 1.08; p < 0.0001)] and a high baseline HbA1c (OR: 1.45; p < 0.0001) received treatment intensification during 180 days.

**Conclusion:**

DPP-4 inhibitors were the most prevalent first-line T2DM treatment followed by metformin in Japan. The findings from this retrospective analysis also support the previously published web survey results and can help understand the real world utilization of T2DM treatment.

*Trial registration* Retrospectively registered

## Background

Type 2 diabetes mellitus (T2DM) is a major health concern that imposes a significant socio-economic burden worldwide. In Japan, the prevalence of T2DM was about 7.7% in adults aged 20–79 years in 2017, and it was among the 10 ten countries in the world with the highest expenditure on diabetes [1]. Despite the availability and advancement of several therapeutic options for the treatment of T2DM [2], less than half of all patients in Japan reach the optimal glycemic goal of glycated hemoglobin (HbA1c) < 7% [3, 4].

Guidelines for T2DM management in Japan provide physicians the discretion to select treatment options based on patient needs and pathophysiology of the disease [2]; this could result in a wide variation in the prescription of antidiabetes drugs (AD) and treatment patterns in clinical practice [5]. Thus, there is a need to understand the factors that drive these preferences in prescriptions.

Furthermore, treatment intensification during the course of the disease is crucial to achieve good glycemic control to prevent diabetes-related complications [6, 7]. In some instances, patients may require an additional oral antidiabetes drug (OAD) in the short term (less than 6 months) after starting the first OAD. However, there is limited evidence on the characteristics of patients who require additional treatment, and there are no data on physicians’ preferences of the OAD as an add-on therapy.

Our earlier results based on an online survey of physicians highlighted the factors influencing the prescribing patterns for drug-naive T2DM patients in Japan [8]. In the present analysis, we used data from The Medical Data Vision (MDV) database, a Diagnosis Procedure Combination (DPC) administrative database containing extensive data from across Japan [9, 10], to help us understand the treatment patterns and factors driving these preferences in the real world setting. Findings from this analysis will provide better understanding of initial treatment patterns and treatment intensification, and factors associated with these prescribing patterns, in drug-naive patients with T2DM in Japan.

## Methods

### Study design and population

**Fig. 1:**
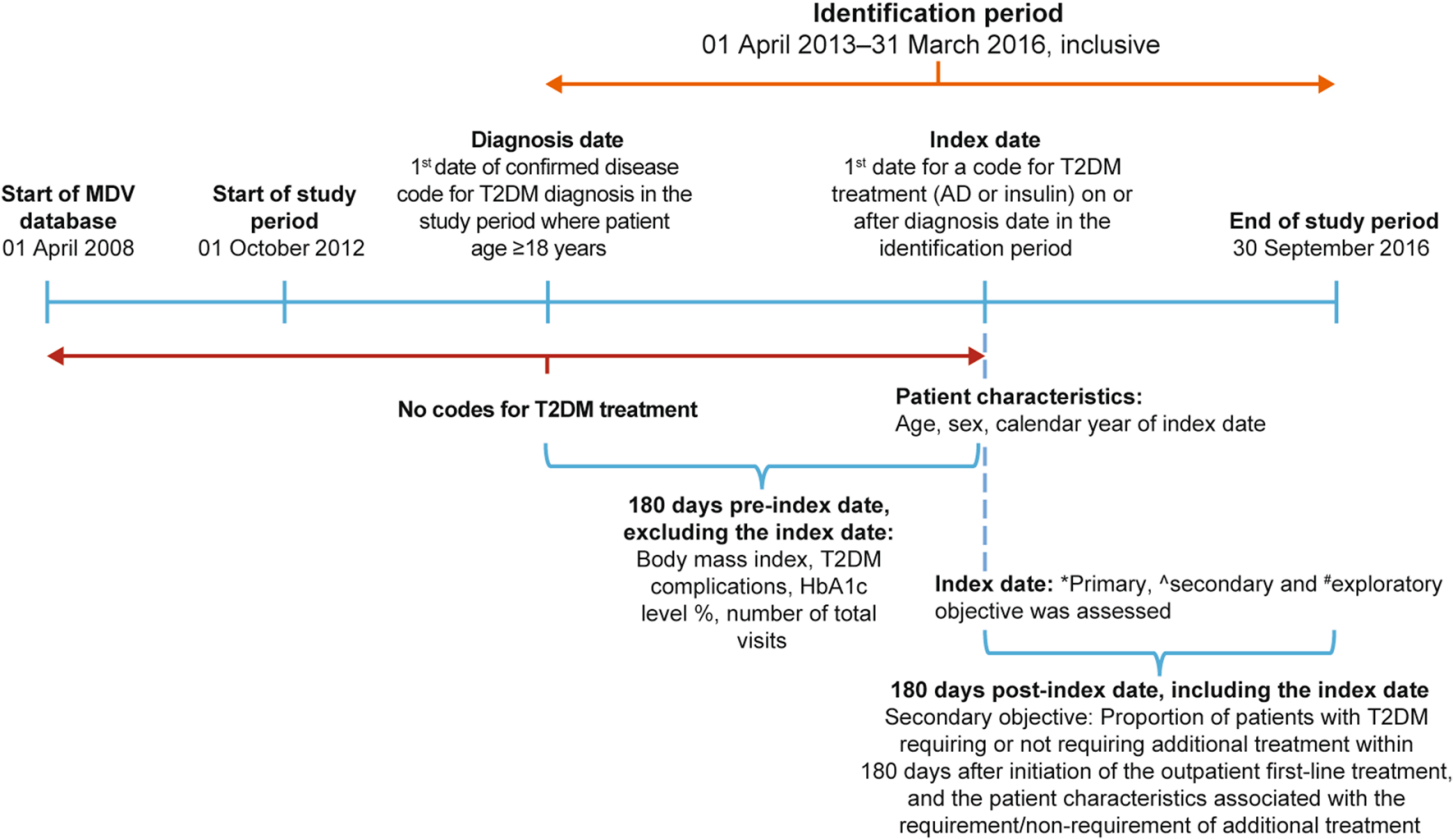
Study schema. *Proportion of patients receiving each type of T2DM therapy (AD or insulin) as first-line treatment. ^^^Features of drug-naive patients treated with the first and second most frequently used outpatient AD monotherapy. ^#^Proportion of patients with T2DM undergoing one or more examinations for diabetic complications of interest. *AD* antidiabetes, *HbA1c* glycated hemoglobin, *MDV* Medical Data Vision, *T2DM* type 2 diabetes mellitus

This was a retrospective cohort, non interventional study involving secondary use of data collected from the MDV database during 01 October 2012 to 30 September 2016 in Japan. Patients aged ≥ 18 years and on the first record with a confirmed diagnosis of T2DM in outpatient settings were included in the analysis. The index date was defined as the date of the first record with a code for T2DM treatment (AD or insulin) on or after the diagnosis date. The pre-index period was 180 days before the index date, while the post-index period was 180 days after the index date. The study schema is depicted in Fig. 1.

### Study objectives

The primary objective of the analysis was to determine the proportion of patients receiving each type of T2DM therapy (AD or insulin) as first-line treatment. The secondary objectives were to determine: (1) the features of drug-naive patients treated with the first and second most frequently used outpatient AD monotherapy, (2) the proportion of patients with T2DM requiring additional treatment within 180 days after initiation of the first-line treatment in outpatient settings, and (3) patient characteristics associated with this additional treatment. In addition, the exploratory objective was to determine the proportion of patients with T2DM undergoing one or more examinations for diabetic complications of interest—neuropathy, retinopathy, and nephropathy.

### Data sources and sample size

The MDV database used in this study is a DPC administrative database. As of 2015, the MDV database included records of > 11 million patients from > 200 acute phase Japanese institutions. Data for elderly patients (> 65 years of age) are included, along with patients’ characteristics (e.g. age, sex, concomitant disease) and treatment information (name and dosage of the prescribed drugs). However, the availability of HbA1c data was limited to approximately 25% of the sampled patients. The coding of diagnoses and disease names was standardized using the International Classification of Diseases, tenth revision (ICD-10) and the disease codes of the Medical Information System Development Center (MEDIS-DC), respectively [11].

### Statistical analysis

Categorical variables were presented as numbers and proportions; continuous variables were expressed as mean, standard deviation, interquartile range, and range. Univariate logistic regression was used to assess the odds ratio (OR) and 95% confidence interval (CI) for the most vs second most frequently prescribed first-line AD with each patient demographic and clinical characteristic of interest. Similar analyses were performed examining patients requiring vs not requiring additional T2DM treatment within 180 days after the index date. The frequency and percentage of missing data were calculated for each variable.

### Ethical considerations

This study was conducted in accordance with the Ethical Guidelines for Medical and Health Research Involving Human Subjects (The Ministry of Education, Culture, Sports, Science and Technology and the Ministry of Health, Labour and Welfare, Japan). A central ethics committee of the Clinical Research Promotion Network reviewed and approved the study protocol since no personally identifiable data was included in the database extraction for the study.

## Results

### Participants

**Fig. 2:**
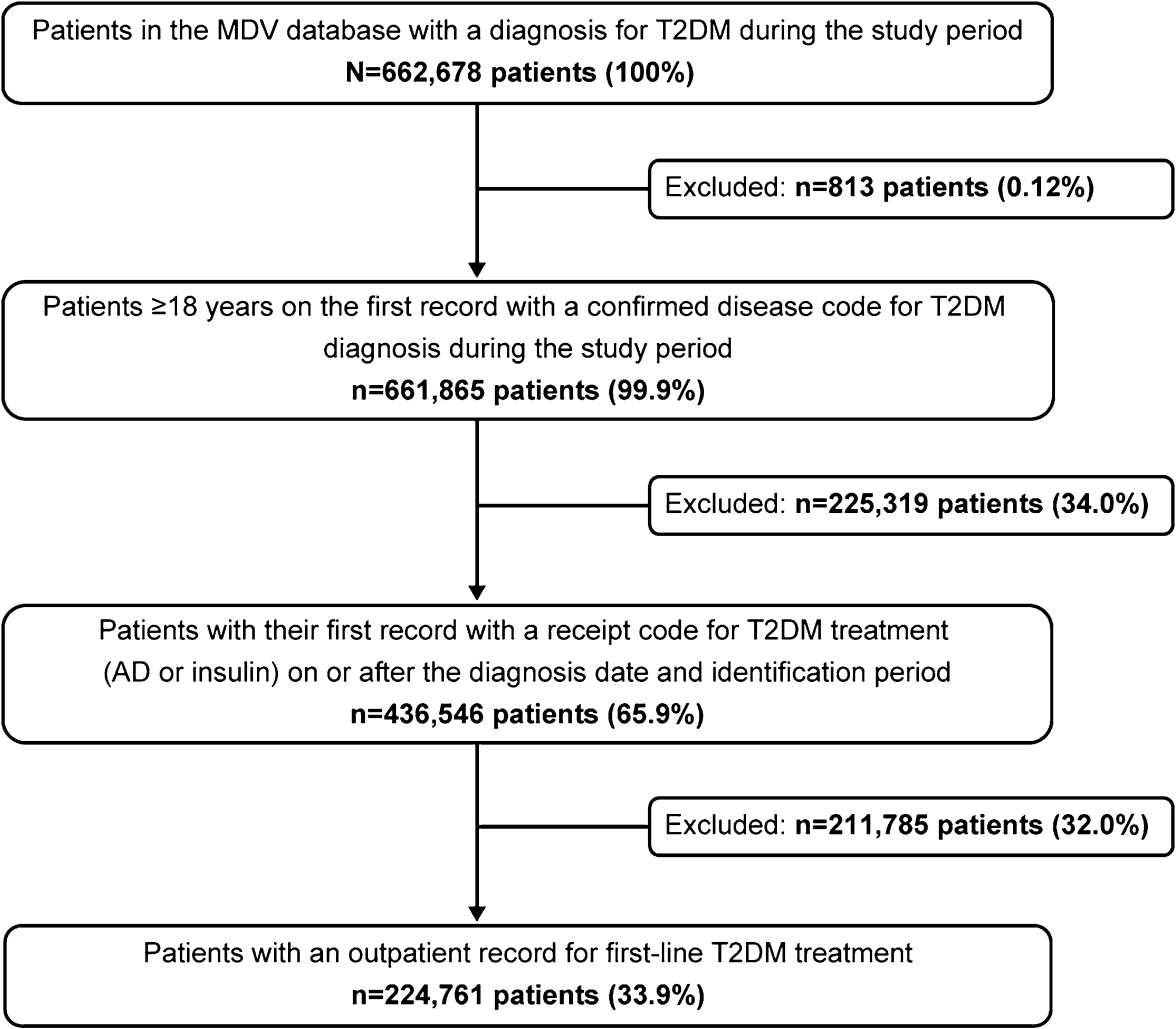
Flow chart of patient selection from the MDV database. *AD* antidiabetes drugs, *MDV* Medical Data Vision, *T2DM* type 2 diabetes mellitus

A total of 662,678 patients in the MDV database with a diagnosis of T2DM during the study period were retrieved for the analysis, of which 436,546 (65.9%) patients were adults with T2DM treatment on or after the diagnosis date. The number of patients with an outpatient record for first-line T2DM treatment was 224,761 (33.9%). Figure 2 is a flowchart depicting the summary of patient selection for the analysis.

### Baseline demographics and clinical characteristics

Details of the baseline demographics and clinical characteristics of the outpatient cohort are presented in Table 1. The mean age of the patients was 65.6 years at the index date and 61.2% were male. The mean body mass index (BMI) was 24.6 kg/m^2^ in the 12,839 (5.7%) patients where data on BMI was available. The proportion of patients diagnosed with coronary heart disease and stroke was the highest at 30.9% followed by liver disease at 21.9%; renal disease was the least diagnosed comorbidity at 9.0%. The mean HbA1c level was 8.0% in the 16,429 (7.3%) patients with available data on HbA1c levels.

**Table 1:** Baseline demographics and clinical characteristics of drug-naive patients with T2DM treated with first-line AD therapy in the outpatient setting

Characteristics	Drug-naive patients (N = 224,761)
Gender (men)	137,391 (61.2%)
Age (years), mean (SD)	65.6 (13.0)
BMI^a^ (kg/m^2^), mean (SD)	24.64 (4.6)
HbA1c level (%)^a^, mean (SD)	7.99 (1.9)
Normal: < 6.0%	746 (0.3%)
Patients with HbA1c level between 6.0% and < 7.0%	4575 (2.0%)
Patients with HbA1c level between 7.0% and < 8.0%	5162 (2.3%)
Patients with HbA1c level ≥ 8.0%	5946 (2.7%)
Comorbidities^a^	
Coronary heart disease and stroke	69,371 (30.9%)
Liver disease	49,199 (21.9%)
Diabetic nephropathy	25,065 (11.2%)
Diabetic retinopathy	24,553 (10.9%)
Diabetic neuropathy	21,147 (9.4%)
Dyslipidemia	129,103 (57.44%)
Hypertension	139, 665 (62.14%)
Renal disease	20,118 (9.0%)
Therapy use	
Antihypertensive drugs	115,576 (51.42%)
Antidyslipidemic drugs	93,863 (41.76%)
Antithrombotic drugs	62,430 (27.78%)
Total visits^a^, mean (SD)	3.8 (5.8)

*AD* antidiabetes, *BMI* body mass index, *HbA1c* glycated hemoglobin, *SD* standard deviation, *T2DM* type 2 diabetes mellitus

^a^Assessed within 180 days before the index date. Values are presented as n (%) unless otherwise stated. % is defined as n/N

Additionally, 2.7% of the outpatients were classified as having diabetes with intensified therapy and HbA1c levels ≥ 8.0%. A total of 91,554 (40.7%) outpatients had at least one clinic visit during 180 days pre-index date, with a mean of 3.8 total visits.

### Selection of first-line AD therapy for drug-naive patients with T2DM

#### Overall

**Fig. 3:**
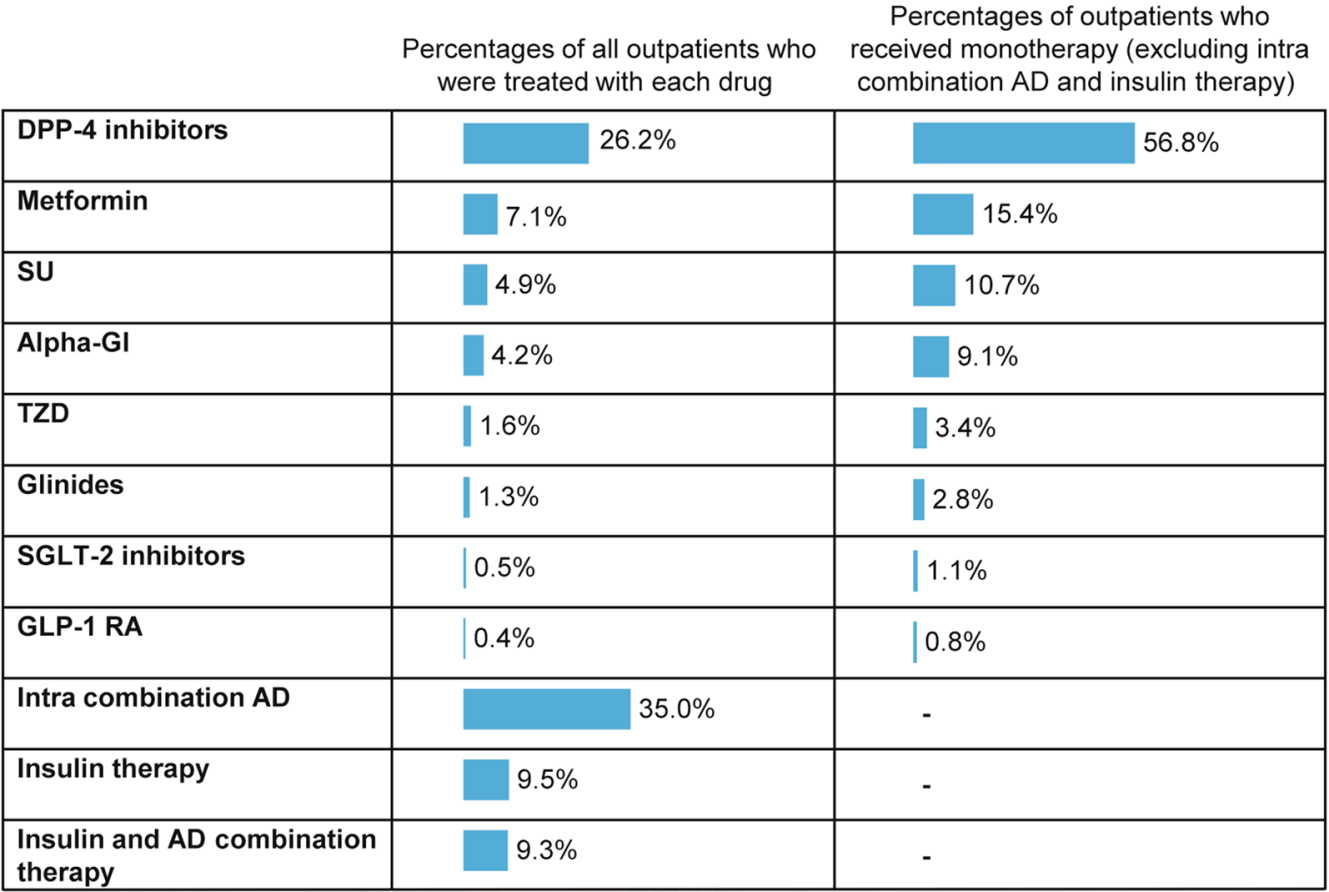
Selection of first-line AD therapy for drug-naive patients with T2DM in the outpatient setting. *AD* antidiabetes drugs, *DPP*-*4* dipeptidyl peptidase-4, *GI* glucosidase inhibitor, *GLP*-*1 RA* glucagon like peptide-1 receptor agonist, *SGLT*-*2* sodium glucose co-transporter-2, *SU* sulfonylurea, *T2DM* type 2 diabetes mellitus, *TZD* thiazolidinediones

Among 436,546 patients included in the drug-naive cohort, 224,761 (51.5%) received their first-line T2DM treatment in the outpatient setting. Of these, 81.2% received AD therapy, 9.3% insulin therapy and 9.5% insulin and AD combination therapy as first-line T2DM treatment at index date. The total proportion of patients who received AD intracombination (35.0%), insulin and AD combination (9.3%) or insulin (9.5%) was 53.8% (Fig. 3). More outpatients received AD intra combination therapy (43.1%) than monotherapy.

#### Monotherapy

Of the 103,789 patients who received monotherapy (excluding patients receiving AD intracombination, insulin plus AD combination and insulin therapy), most were treated with dipeptidyl peptidase-4 inhibitors (DPP-4 inhibitors; 56.8%), followed by metformin (15.4%), sulfonylurea (SU; 10.7%), alpha-glucosidase inhibitors (alpha-GI; 9.1%), thiazolidinediones (3.4%), glinides (2.8%), sodium glucose co-transporter-2 inhibitors (SGLT2i; 1.1%) and glucagon like peptide-1 receptor agonists (0.8%) (Fig. 3).

### Patient characteristics associated with the selection of DPP-4 inhibitors (most prevalent first-line AD) versus metformin (second most prevalent first-line AD) in the outpatient setting

Patients diagnosed with renal disease, coronary heart disease and stroke were 4.20 (p < 0.0001) and 2.22 (p < 0.0001) times more likely to be prescribed DPP-4 inhibitors as first-line outpatient treatment for T2DM. Metformin was more likely to be initiated in patients with diabetic retinopathy, diabetic neuropathy, and diabetic nephropathy, the odds of which were 0.74 (p < 0.0001), 0.94 (p = 0.16) and 0.96 (p = 0.22), respectively. With every 1 year increase in age at the index date, the odds of being prescribed DPP-4 inhibitors was 1.06 times (p < 0.0001) more likely than metformin. With every 1 kg/m^2^ increase in BMI and 1% increase in HbA1c level, metformin was more likely to be prescribed as the first-line AD therapy vs DPP-4 inhibitors, with odds of 0.90 (p < 0.0001) and 0.83 (p < 0.0001), respectively (Table 2).

**Table 2:** Univariate logistic regression analysis of DPP-4 inhibitors vs metformin as first-line outpatient AD therapy with patient characteristics and comorbidities

Prescribed drug class	Treatment factors	OR	95% CI	
			Lower limit	Upper limit
DPP-4 inhibitors	Renal disease^a^	4.20*	3.82	4.63
	Coronary heart disease and stroke^a^	2.22*	2.13	2.32
	Patient total visits^a^	1.34*	1.28	1.41
	Male vs female^b^	1.10*	1.06	1.14
	Age at the index date	1.06*	1.05	1.06
Metformin	Liver disease^a^	0.96^§^	0.92	1.00
	Diabetic nephropathy^a^	0.96^#^	0.90	1.03
	Diabetic neuropathy^a^	0.94^#^	0.87	1.03
	BMI^a^	0.90*	0.89	0.92
	HbA1c level, %^a^	0.83*	0.80	0.86
	Diabetic retinopathy^a^	0.74*	0.70	0.79

An OR > 1 indicates DPP-4 inhibitors were prescribed over metformin with the independent variable, and vice versa

*AD* antidiabetes drugs, *BMI* body mass index, *CI* confidence interval, *DPP*-*4* dipeptidyl peptidase-4, *HbA1c* glycated hemoglobin, *ns* non significant, *OR* odds ratio

* p < 0.0001, ^§^p = 0.044, ^#^p = ns

^a^Assessed within the 180 days before the index date

^b^Female were the referent group for male vs female. Patient’s total visits > sample median total visits vs patient’s total visits ≤ sample median total visits

### Patient characteristics associated with additional T2DM treatment during 180 days post-index date

Of the 224,761 patients who received their first-line T2DM treatment in the outpatient setting, 44,951 (20.0%) required additional T2DM treatment within 180 days post-index date. The factors positively and negatively associated with the requirement of additional treatment during 180 days post-index date are presented in Table 3. Male were 1.03 times more likely to receive additional T2DM treatment compared to female (p = 0.0026). The odds of not being prescribed additional T2DM treatment during 180 days post-index date increased by 1.02 times with every 1 year increase in age at the index date (OR: 0.98; p < 0.0001). There was no association between BMI and additional treatment in the outpatient cohort.

**Table 3:** Patient characteristics associated with additional treatment during 180 days post-index date

Patient characteristics	OR	95% CI	
		Lower limit	Upper limit
HbA1c level %^a^	1.45*	1.42	1.48
Diabetic retinopathy^a^	1.33*	1.29	1.37
Patient total visits^a^	1.18*	1.14	1.22
Diabetic nephropathy^a^	1.08*	1.04	1.11
Diabetic neuropathy^a^	1.05^§^	1.02	1.09
Gender (male vs female^b^)	1.03^^^	1.01	1.06
BMI^a^	1.00^#^	0.99	1.01
Age at index date	0.98*	0.98	0.98
Renal disease^a^	0.94^ψ^	0.91	0.98
Liver disease^a^	0.88*	0.85	0.90
Coronary heart disease and stroke^a^	0.73*	0.72	0.75

An OR > 1 indicates a positive association with receiving additional treatment

*BMI* body mass index, *CI* confidence interval, *HbA1c* glycated haemoglobin, *ns* non significant, *OR* odds ratio

* p < 0.0001, ^§^p = 0.0037, ^^^p = 0.0026, ^ψ^p = 0.0014, ^#^p = 0.721 (ns). Patient’s total visits > sample median total visits vs patient’s total visits ≤ sample median total visits

^a^Assessed within the 180 days before the index date

^b^Female were the referent group for male vs female

Patients diagnosed with renal disease except for diabetic nephropathy, liver disease and coronary heart disease and stroke were 0.94 (p = 0.0014), 0.88 (p < 0.0001) and 0.73 (p < 0.0001) times less likely to receive additional treatment, respectively, compared to patients not diagnosed with these conditions. In contrast, patients with diabetic retinopathy, neuropathy, and nephropathy were 1.33 (p < 0.0001), 1.05 (p = 0.0037), and 1.08 (p < 0.0001) times more likely to receive additional treatment, respectively.

Patients with more than the sample median number of total visits were 1.18 times more likely to be prescribed additional T2DM treatment during the 180 days post-index date (p < 0.0001).

### Examination of diabetic complications of interest

**Fig. 4:**
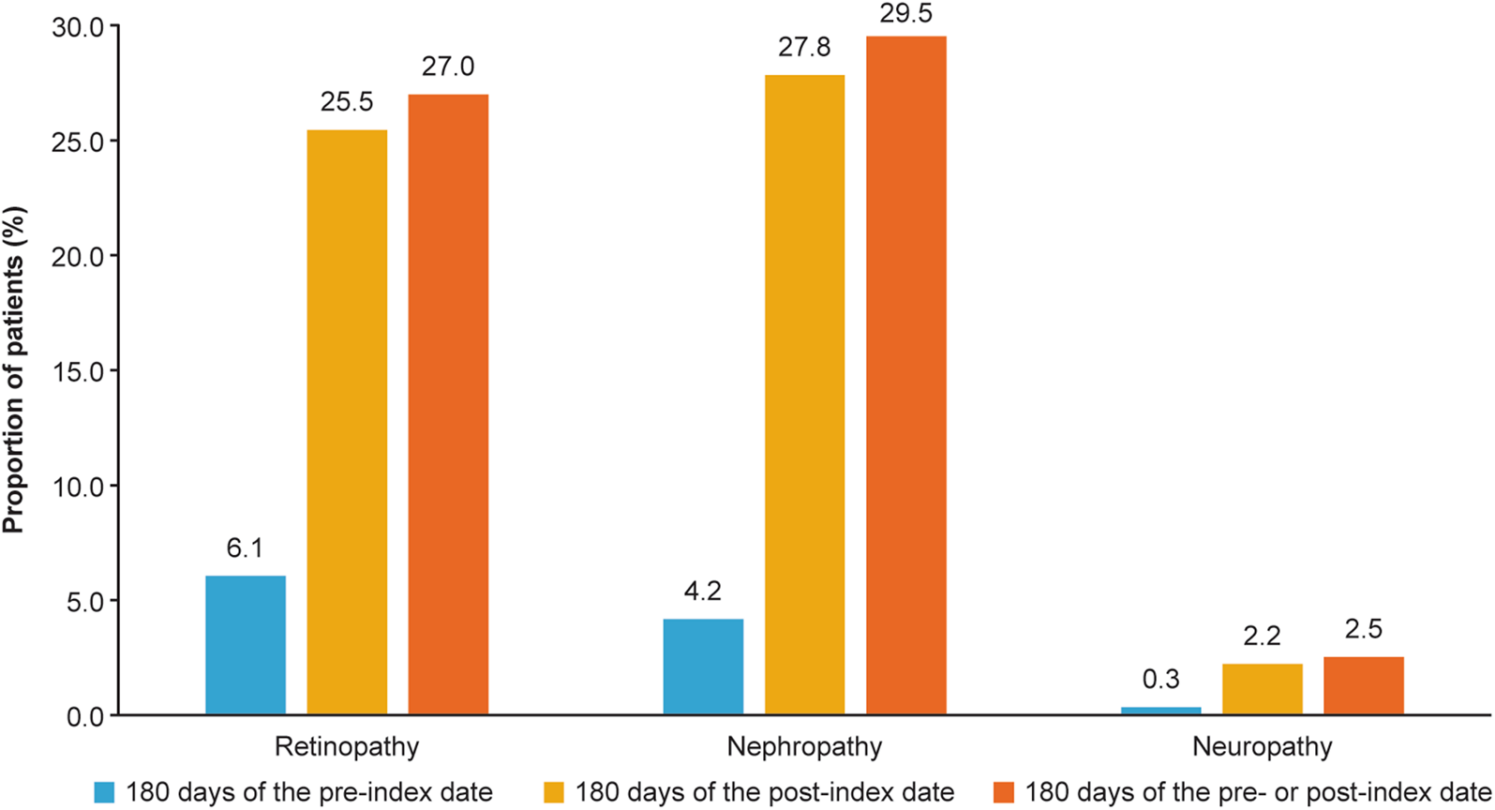
Proportion of patients with an examination for diabetic complications

The proportion of patients with an examination for diabetic retinopathy and nephropathy were 27.0% and 29.5%, respectively, during the 180 days pre-index or post-index date (Fig. 4). The proportion of patients with an examination for neuropathy was negligible (2.5%). The proportion of patients with an examination for each of the three diabetic complications was higher during the 180 days of the post-index date compared to pre-index period.

## Discussion

The database analysis revealed the most frequently used AD therapy in patients with T2DM treated in the outpatient setting, characteristics of these patients associated with the choice of treatment, as well as with additional treatment for T2DM during 180 days post-index treatment. The results showed that DPP-4 inhibitors were the most prevalent outpatient AD monotherapy followed by metformin, which are in agreement with the findings from the web-based survey of physicians’ prescribing preferences [8] as well as other studies [12]. In a recent study based on the data from two large, administrative claims databases, Japan Medical Data Center (JMDC) and MDV, the most common index prescription was for DPP-4 inhibitors as monotherapy (JMDC: 44.0%; MDV: 54.8%), followed by biguanide, insulin and combination therapy. Additionally, in untreated patients whose index prescription was for any other antidiabetes drug class, DPP-4 inhibitors were the most frequently used add-on, treatment switch and combination regimen [13].

The launch of DPP-4 inhibitors in the Japanese market in 2009 and the increase in the daily permissible prescription dose of metformin from 750 to 2250 mg in 2010 are plausible reasons for this trend of prescriptions [14]. In Western countries, however, metformin is mostly used as a first-line treatment for T2DM, as recommended by the American Diabetes Association (ADA) and the European Association for the Study of Diabetes (EASD) [15, 16].

DPP-4 inhibitors and metformin were the 2 most frequently prescribed AD treatments in both the web survey and database analysis. The proportion of patients prescribed metformin in the database analysis was only slightly higher than those prescribed with SU and alpha-GI, which differs from the results of the web survey. One of the possible reasons could be that there may be several patients who initiated treatment in other clinics and were later shifted to the present hospital with high number of diabetes specialists. This may also be the reason for the high usage of SU monotherapy, as the clinic physicians, mainly generalists might have preferred SU at that time. Nevertheless, usage of metformin was still low compared to the findings from the previous web-based survey [8], indicating a gap between the physicians’ intentions and the actual prescription pattern in the real-world setting in Japan. To optimize the usage of metformin, there is a need to discuss and overcome the barriers, which may lead to hesitation among physicians to prescribe it.

Overall, the proportion of patients diagnosed with coronary heart disease/stroke was comparatively higher as compared to those with other comorbidities in the drug-naive cohort at baseline. One of the factors could be considerable proportion of patients with hypertension and dyslipidemia, which are established risk factors for cardiovascular and liver diseases [17, 18]. Another plausible reason could be that data from DPC hospitals also included patients aged > 65 years possibly with multiple moderate-to-severe comorbidities [19, 20] which could have resulted in higher proportion of patients with coronary heart disease/stroke or liver diseases.

It was observed that elderly patients and patients with renal disease were associated with a prescription of DPP-4 inhibitors as the index treatment. This may be partly due to the use of metformin being avoided in patients at risk for lactic acidosis, such as older individuals and those with advanced renal insufficiency [21]. Thus, DPP-4 inhibitors may be perceived as the safer treatment option in such cases. Furthermore, BMI was associated with the prescription of metformin as the index T2DM treatment vs DPP-4 inhibitors, confirming the results of the web-based survey [8]. This may be supported by the findings from the UKPDS 34 study, where metformin was shown to decrease the risk of diabetes-related complications in overweight patients and was associated with weight neutrality and fewer hypoglycemic events [22]. However, there are studies such as the Melbin observational research (MORE) study in Japanese patients which demonstrated that the HbA1c reduction was comparable (0.9 ± 1.2% vs 1.0 ± 1.4%) in patients with BMI ≥ or < 25 kg/m^2^ indicating that regardless of body weight, metformin may have the same effect in patients with T2DM [23].

The current results showed that a diagnosis of macrovascular disease and renal disease were associated with prescription of DPP-4 inhibitors rather than metformin, while diagnosis of a microvascular complication was associated with the prescription of metformin over DPP-4 inhibitors as the index T2DM treatment. This may indicate that metformin was considered more useful for patients diagnosed with microvascular disease based on the findings from the UKPDS study [22]. However, the reasons for the use of DPP-4 inhibitors over metformin in patients with coronary heart disease and stroke remains unclear. Additionally, at that time of the database analysis, DPP-4 inhibitors were considered to increase hypoglycemia, as indicated in several reports of hypoglycemia with DPP-4 inhibitors in combination with SUs [24]. As low glycemia is one of the causes of retinopathy, metformin may have been preferred over DPP-4 inhibitors in patients with microvascular disease. Findings from earlier studies suggest that about 1 in 3 patients with T2DM will develop retinopathy, 1 in 4 will develop nephropathy, and 1 in 2 will develop neuropathy [25–27] due to suboptimal glycemic control. However, with optimal glycemic control, these microvascular complications may be delayed or prevented [28, 29].

The present database analysis showed that high baseline HbA1c was the key driving factor influencing physicians to choose add-on treatment, while BMI did not affect the decision of treatment intensification. The proportion of patients diagnosed with macrovascular disease requiring additional T2DM treatment during 180 days post-index date was markedly low while the proportion of patients diagnosed with microvascular disease requiring additional treatment was comparatively high. This may be attributed to the insights from the ACCORD study, which showed that intensive glucose lowering therapy does not have an effect on the prevention of death for those who already have macrovascular disease [30]. In such a condition, the physician’s concern might be more towards avoiding low blood glucose than in lowering HbA1c. On the other hand, studies have demonstrated that intensive glucose-lowering therapy reduces or delays the onset and progression of diabetic retinopathy, neuropathy and nephropathy [28, 29, 31].

In the present database analysis, patients received more examinations for diabetic retinopathy and nephropathy compared to diabetic neuropathy. The clinical evaluation and investigations of diabetic neuropathy involve challenges and its diagnosis can be difficult due to the co occurrence of other similar symptoms, thus only a small proportion of patients are extensively evaluated for this condition [32]. As most of these examinations should have been conducted before treatment initiation, it is possible that several patients included in the current analysis may have been introduced from another hospital.

Certain limitations of this study are inherent to all studies using secondary data [33] and must be acknowledged. The MDV database has several limitations, the most important being the inability to follow the clinical record if the patient moves from one hospital to another. Hence, there is a possibility that patients were misclassified as drug-naive since we could not, from this database, differentiate real drug-naive patients and patients referred from clinics who have already been treated with AD. Further, the analysis was not adjusted for key confounding factors such as age, gender, duration of diabetes, and thus future studies including multivariate logistic regression models to simultaneously control multiple potential confounders are warranted. Additionally, patients included in the analysis were a convenience sample from hospitals contributing data to the database rather than a random sample of patients in order to meet the selection criteria. Although we cannot be sure that our convenience sample is representative of the general T2DM patient population in Japan, the MDV database includes extensive patient specific data from over 100 acute phase hospitals in Japan [9, 10].

## Conclusions

The present database analysis confirms that the most prevalent first-line outpatient T2DM monotherapy is DPP-4 inhibitors in Japan, followed by metformin. The dominant patient characteristic associated with receiving a prescription of a DPP-4 inhibitor over metformin was a diagnosis of renal impairment. Male compared to female, patients with diabetic complications and high baseline HbA1c, were more likely to receive treatment intensification. Although these analyses did not adjust for key confounders, they may offer some early evidence to assist physicians with understanding patient characteristics associated with the initiation of T2DM treatment and additional therapy. The findings from the web survey and database analysis revealed similarities as well as differences between physicians’ intentions and the actual prescription of drugs among drug-naive T2DM patients in Japan. Overall, these findings may help physicians understand real world utilization of T2DM treatment among Japanese adults and further help refine treatment algorithms.

## Data Availability

The data sets generated during and/or analyzed during the current study are available from the corresponding author on reasonable request.

## Abbreviations

AD: antidiabetes drugs
ADA: American Diabetes Association
Alpha-GI: alpha-glucosidase inhibitor
BMI: body mass index
DPC: Diagnosis Procedure Combination
DPP-4: dipeptidyl peptidase-4
EASD: European Association for the Study of Diabetes
ICD-10: International Classification of Diseases, tenth revision
MDV: Medical Data Vision
MEDIS-DC: Medical Information System Development Center
MORE: Melbin observational research
OAD: oral antidiabetes drug
SGLT2i: sodium glucose co-transporter-2 inhibitor
SU: sulfonylurea
T2DM: type 2 diabetes mellitus
